# *Heracleum sosnowskyi* Manden. in the Context of Sustainable Development: An Aggressive Invasive Species with Potential for Utilisation in the Extraction of Furanocoumarins and Essential Oils

**DOI:** 10.3390/jox16010006

**Published:** 2026-01-01

**Authors:** Ekaterina Sergeevna Osipova, Evgeny Aleksandrovich Gladkov, Dmitry Viktorovich Tereshonok

**Affiliations:** K. A. Timiryazev Institute of Plant Physiology, Russian Academy of Sciences, 127276 Moscow, Russia; gladkovu@mail.ru (E.A.G.); diman_ter_vi@mail.ru (D.V.T.)

**Keywords:** *Heracleum sosnowskyi*, *H. sosnowskyi*, hogweed, furanocoumarins, essential oils, invasive species, elimination with utility

## Abstract

*Heracleum sosnowskyi* Manden., or *H. sosnowskyi*, of the Apiaceae was first cultivated in the USSR in 1947 as a potential fodder plant. Due to the development of cold-resistant cultivars and the characteristics of *H. sosnowskyi*, it quickly became feral. As a result, *H. sosnowskyi* began to spread as an aggressive invasive species in the 1970s and 1980s. By the 90s it had become an ecological disaster. As well as forming monocultures and displacing native species, *H. sosnowskyi* contains furanocoumarins, photosensitizing compounds that increase skin sensitivity to ultraviolet rays and cause severe burns. In addition, furanocoumarins have cytotoxic, genotoxic, mutagenic and estrogenic effects. *H. sosnowskyi* also contains essential oils, which are particularly active during flowering and can irritate the mucous membranes of the eyes and respiratory tract, as well as cause allergic reactions in the form of bronchospasm in people with asthma and hypersensitivity. When released in high concentrations, these biologically active compounds have an allelopathic effect on native plant species, displacing them and reducing biodiversity. As *H. sosnowskyi* is not native; the biologically active compounds it secretes have a xenobiotic effect, causing serious damage to the ecosystems it occupies. However, in parallel with these negative properties, furanocoumarins have been found to be effective in the treatment of cancer and skin diseases. Furanocoumarins possess antimicrobial antioxidant osteo- and neuroprotective properties. Essential oils containing octyl acetate, carboxylic acid esters, and terpenes can be used in the pharmaceutical industry as antiseptic and anti-inflammatory agents. Additionally, essential oils can be used as biofumigants and natural herbicides. A comprehensive approach allows *H. sosnowskyi* to be viewed in two ways. On the one hand, it is an aggressive alien species that causes significant damage to ecosystems and poses a threat to human health. On the other hand, it is a potentially valuable natural resource whose biomass can be used within the principles of the circular economy. It is hoped that the use of *H. sosnowskyi* for economic interests can be a partial compensation for the problem of its aggressive invasion, which is of anthropogenic origin.

## 1. Introduction

*Heracleum sosnowskyi* Manden. (hogweed) was first described in Georgia in 1944 by I. P. Mandenova. It was named after her teacher, the Caucasian flora researcher D. I. Sosnovsky, and classified as belonging to the *Heracleum* genum, section *Pubescentia* [1]. The description of the plant was published during the war years, and soon after the Soviet Union’s victory, the country was in dire need of agricultural recovery. The country required an abundant and inexpensive feed source for livestock. *H. sosnowskyi* seemed to be the most suitable for this purpose [2]. It was highly productive and adaptable, had a short vegetation period and was adapted to short day-light hours. It was also resistant to pests and low temperatures, grew on poor soils and had similar protein content to silage crops such as maize and lucerne [3,4,5]. However, it soon became clear that contact with the skin followed by exposure to sunlight caused severe photodermatitis. Furthermore, cows that consumed silage containing a high proportion of *H. sosnowskyi* produced milk with an unpleasant taste and, in some cases, infertility was observed. The plant “escaped” from the fields and began to invade the local flora [6]. Research into the chemical composition of *H. sosnowskyi* began after it was introduced to agriculture. In the Soviet Union, research was conducted at the All-Russian Scientific Research Institute of Medicinal and Aromatic Plants, the Komarov Botanical Institute of the Russian Academy of Sciences, the Kharkiv Pharmaceutical Institute and other research institutes in Belarus, Latvia, Uzbekistan and the Transcaucasian republics. Chemical analysis showed that furanocoumarins are responsible for the photosensitizing, mutagenic, and allelopathic properties of *H. sosnowskyi*. During those years, many reference books and publications on furanocoumarins were released, and most of their structures were identified [6,7,8,9,10]. The composition and chemical properties of *H. sosnowskyi* essential oil were carefully studied [11]. *H. sosnowskyi* essential oil is characterised by its high concentration of volatile, biologically active compounds. Its composition is dominated by esters of carboxylic acids (octyl acetate) andesters of aliphatic alcohols (hexyl 2-methylbutanoate, hexyl butanoate), aldehydes (decanal, octanal), and alcohols (octanol). Terpenes (γ-terpinene and α-pinene) are present in smaller quantities. Aldehydes and terpenes are known to be potential allergens and irritants. They can cause contact dermatitis and irritation of the mucous membranes and respiratory tract, particularly in sensitive individuals, due to their pronounced irritating and toxic activity. *H. sosnowskyi* pollen can cause allergic reactions [12,13]. High concentrations of essential oil in the environment also have an inhibitory effect on the germination and growth of other plants. In a study by Mishyna et al. (2015), the allelopathic properties of octanal were proven [14]. Biologically active compounds released by *H. sosnowskyi*, which accumulate at high concentrations, represent xenobiotic factors for ecosystems of the European part of Russia. These compounds disrupt established biotic interactions and the stability of natural communities, as well as having an adverse effect on human health. Furthermore, populations of *H. sosnowskyi* in northern regions appear to synthesise more toxic biologically active compounds in response to stressful climatic and environmental conditions [15]. These compounds contribute significantly to the establishment of hogweed as an aggressive invasive plant, giving it competitive advantages in the ecosystems it colonises. Today, *H. sosnowskyi* is considered one of the most aggressive invasive plants, and one method of control is mowing. Mowing produces large quantities of biomass that need to be disposed of. The most effective method of disposal is to extract valuable compounds for practical use [16]. This review aimed to identify recent studies on the extraction of furanocoumarins and essential oils from *H. sosnowskyi*. This process reduces the abundance of the plant, thereby decreasing its negative environmental impact, while also having the potential for application in pharmacology, biotechnology, the chemical industry and agriculture.

## 2. Toxic Effects of *H. sosnowskyi* Are Intensified Under More Northern Climatic Conditions

The first studies of *H. sosnowskyi* in the USSR were conducted at the Polar-Alpine Botanical Garden in the Kola Peninsula, established in 1931 on the initiative of Avrorin [17]. In 1946, Marchenko A.A. was assigned to carry out comparative testing of several perennial grasses transplanted by the Botanical Garden in order to identify the most promising species for silage production. *H. sosnowskyi* showed the highest levels of vegetative biomass accumulation [18]. Marchenko did not conceal the irritating properties of *H. sosnowskyi*; in his literature review he cited works [19,20,21] describing cases of poisoning in livestock and dermatitis in humans. Nevertheless, the experience of local collective farms was positive: silage made from *H. sosnowskyi* was successfully used as cattle feed, and in early spring, animals readily consumed young shoots. Marchenko also conducted feeding experiments on animals which revealed no deviations from normal physiological parameters. Moreover, the test group showed an increase in milk yield. Under natural conditions in the central and eastern regions of the Caucasus, *H. sosnowskyi* did not exhibit such pronounced toxicity. The toxic effects of *H. sosnowskyi*, which cause photodermatitis in humans and feed poisoning in animals, are generally associated with compounds from various chemical classes, including coumarins, terpenes, saponins, alkaloids and flavonoids. It has been established that the chemical composition of *H. sosnowskyi* is strongly influenced by habitat conditions, the phase of vegetation, and weather factors [6,11]. In response to stress factors typical of harsher climates (e.g., the forest zones of the European part of Russia), the plant synthesises greater amounts of defensive secondary metabolites [22]. In northern populations, both the composition and concentration of coumarins and furanocoumarins change: their molecules become more oxygenated and contain methoxy, hydroxy, oxide, and ester groups, which increase overall toxicity [23]. Furthermore, mutagenic activity and DNA-damaging potential have been shown to vary depending on the combination of furanocoumarins present [24,25]. The essential oils of *H. sosnowskyi* populations growing in northern latitudes also contain terpenes absent in plants collected from natural Caucasian populations [26]. Taken together; these factors likely explain the contradictory data on the toxicity of *H. sosnowskyi*, as well as the ongoing debate regarding their suitability as green fodder, hay, or silage [27]. The experience of introducing Sosnowski’s hogweed demonstrates that, before introducing new species, thorough and comprehensive environmental studies must be conducted over a long period of time, with mandatory dynamic monitoring. Only then can a decision be made about their large-scale use.

## 3. The Negative Impact of *H. sosnowskyi* on the Environment Is One of the Priority Interregional Environmental Problems

**Figure 1:**
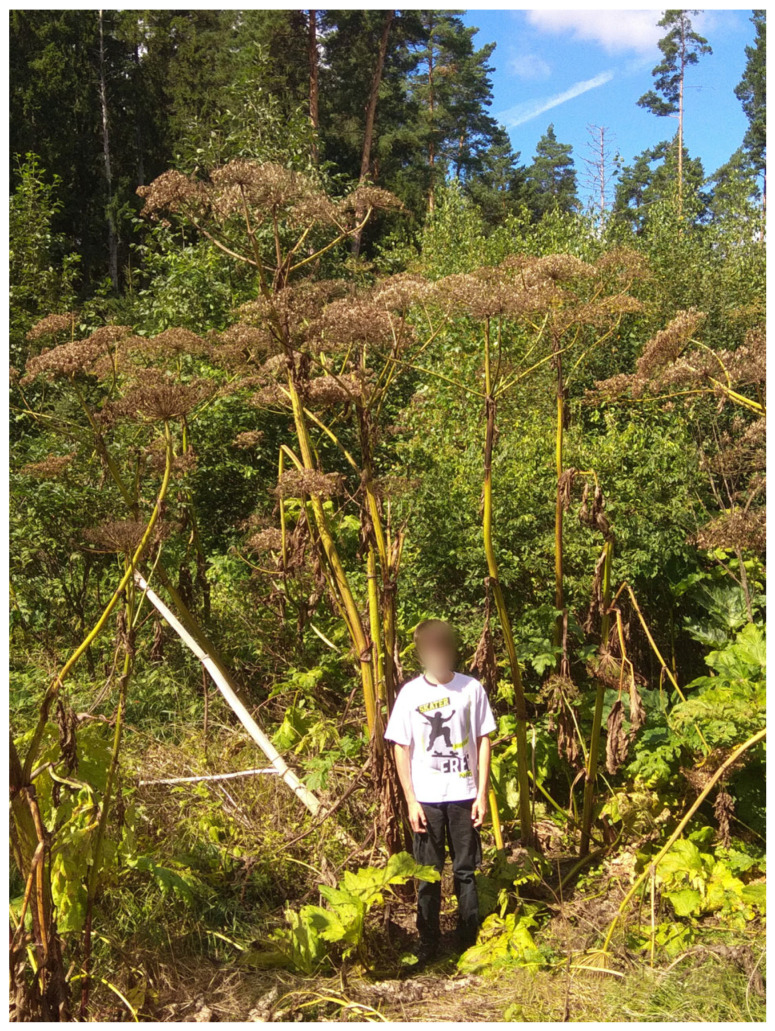
*H. sosnowskyi* on the side of the road (Mozhaisky District, Russia. The person near hogweed is 170 cm tall).

At present, *H. sosnowskyi* is one of the most aggressive invasive species in the temperate zone of Europe. Its northern distribution limit is defined by the short, cold summers of the Kola Peninsula and the Arkhangelsk region, while the southern limit is constrained by the droughts of the forest–steppe and steppe zones. The western range has expanded into the Baltic States, Poland, and Belarus, and the eastern limit extends to the Pre-Urals and Western Siberia. The species rapidly colonises disturbed and unused areas such as roadsides (Figure 1), abandoned fields, riverbanks, and forest clearings [28]. The most features of *H. sosnowskyi* invasiveness include exceptionally high seed productivity (up to 100,000 seeds per plant), efficient seed dispersal over long distances, high germination rates, long-term storage in the soil for up to 10 years [29,30], and the ability to use natural resources faster than native species [31]. *H. sosnowskyi* is also capable of hybridising both with other giant *H. sosnowskyi* species [6] and with native species. In the Konakovo district (Russia), a hybrid of *H. sosnowskyi* and *H. sibiricum* has been recorded [32]. *H. sosnowskyi* is characterised by a high level of genetic diversity, which determines its significant genetic plasticity and ability to quickly adapt to various climatic and environmental conditions [33]. *H. sosnowskyi* releases biologically active compounds into the environment that exert allelopathic effects, inhibiting seed germination, growth, and development of native plant species, while also altering soil composition and soil microbiota [34,35,36]. As a result, *H. sosnowskyi* actively displaces indigenous vegetation. This, on the one hand, leads to a reduction in biodiversity, and on the other hand, depletes the forage base, consequently reducing the populations of birds or herbivorous animals associated with traditional meadow and riparian ecosystems [37]. In new habitats, *H. sosnowskyi* has virtually no natural enemies or population regulators, which, combined with its high fecundity and tolerance to adverse conditions, provides a significant competitive advantage and facilitates rapid spread. The mass proliferation of *H. sosnowskyi* goes beyond a local ecological problem and becomes a serious interregional threat, affecting biodiversity, agriculture, and public health. Legislative measures to control *H. sosnowskyi* in Russia began in 2012, when it was removed from the list of forage crops and product classifiers, officially recognising it as a weed [38]. Starting in 2025, legislation imposes an obligation to control *H. sosnowskyi* and other hazardous alien (invasive) plants for all landowners, tenants, and users, including agricultural, garden, forest, and municipal lands. Non-compliance may result in administrative fines and potential land seizure [39].

## 4. *H. sosnowskyi* in the Context of Sustainable Development

One of the fundamental principles of sustainable development is the protection and restoration of ecosystems, combating climate change, preserving biodiversity, and promoting rational consumption and production patterns. The only way to protect biodiversity in ecosystems colonised by *H. sosnowskyi* is to eradicate the invasive species completely. The methods currently used to combat *H. sosnowskyi* are not very effective. Most of the measures are based on the use of herbicides, which are themselves environmentally hazardous substances and have significant restrictions on their use, especially near water bodies, populated areas, and specially protected natural areas [40]. From the perspective of sustainable development, it seems more appropriate to use environmentally safe methods to destroy *H. sosnowskyi*, such as regular mowing, followed by the rational use of the resulting biomass [16]. The content of protein and sugars in the dry matter of *H. sosnowskyi* varies with the harvesting season, ranging from 13.4 to 16.6% and from 20.7 to 25.8%, respectively [41]. The above-ground mass of *H. sosnowskyi* contains many vitamins, zinc, copper, manganese, iron and sufficient calcium, as well as other microelements [42]. In addition to their negative properties for humans, furanocoumarins (bergamot, xanthotoxin, psorolin, isopimpenilin, and angelicin, etc.) have a number of positive ones: antitumour [43,44], antioxidant [45], osteoporotic [46], antibacterial, and antifungal effects [47,48]. Flavonoids and phenolic acids found in *H. sosnowskyi* have anti-inflammatory properties [49,50]. By reducing background inflammation, polyphenols improve insulin sensitivity, protect neurons, and inhibit antigen genesis and cancer cell proliferation [51]. In addition, flavonoids (rutin) and phenolic acids (chlorogenic acid) [52] modulate immune system activity, which may explain the traditional use of *H. sosnowskyi* decoctions in folk medicine for alleviating arthritis, rheumatism, skin inflammation, and burns. The essential oil of *H. sosnowskyi* is composed mainly of aliphatic compounds such as esters (e.g., octyl acetate), alcohols (e.g., octanol), and aldehydes (e.g., octanal, trans-2-hexenal) along with terpenes and terpenoids, including α-pinene and p-cymene. This complex composition results in a wide range of biological activities, including antiseptic, antiviral, antispasmodic and expectorant effects [11]. Pectin polysaccharides and arabinogalactan proteins isolated from *H. sosnowskyi* [53] exhibit anti-inflammatory, immunostimulating and enterosorbent properties, normalising digestion [54,55]. *H. sosnowskyi* is a source of a wide range of biologically active substances, which could be useful for the economy [56,57,58]. However, there are a number of significant limitations associated with this use. One of the issues with processing *H. sosnowskyi* is that the available biomass is finite. As noted above, the cultivation of *H. sosnowskyi* is prohibited by federal law, which excludes the possibility of growing it on an industrial scale and exacerbating environmental problems. Nevertheless, the area covered by natural *H. sosnowskyi* stands in the European part of Russia currently accounts for 15% of natural landscapes, with an annual growth rate of 10% [58,59,60]. This indicates that the volume of biomass formed by wild populations of hogweed may be sufficient to implement programmes for its phased utilisation and processing over a decade, without the need for artificial reproduction of the plant. Anotherissue with processing *H. sosnowskyi* is the significant variability in the composition and concentration of the target products, furanocoumarins and essential oil, which makes it difficult to standardise the technological process. However, in the context of combating invasive species, processing can be viewed not as commercial production, but as a means of utilising biomass, which additionally allows valuable substances to be obtained. The creation of waste-free technological chains is a promising direction. For example, the cellulose residue formed after the extraction of biologically active compounds can be used to produce fuel pellets or cellulose [61]. This solution would significantly reduce the volume of harmful production waste. To successfully combat *H. sosnowskyi*, effective government programmes are needed to map and monitor the spread of invasive species, restore damaged ecosystems, reclaim land and encourage agricultural use of abandoned land. The processing of invasive hogweed thickets can become an element of sustainable natural resource management. This combines environmental safety with practical benefits. But its effectiveness depends on a government support and systematic approach.

## 5. *H. sosnowskyi* Is a Source of Furanocoumarin

### 5.1. Characteristics of Furanocoumarin in H. sosnowskyi

**Figure 2:**
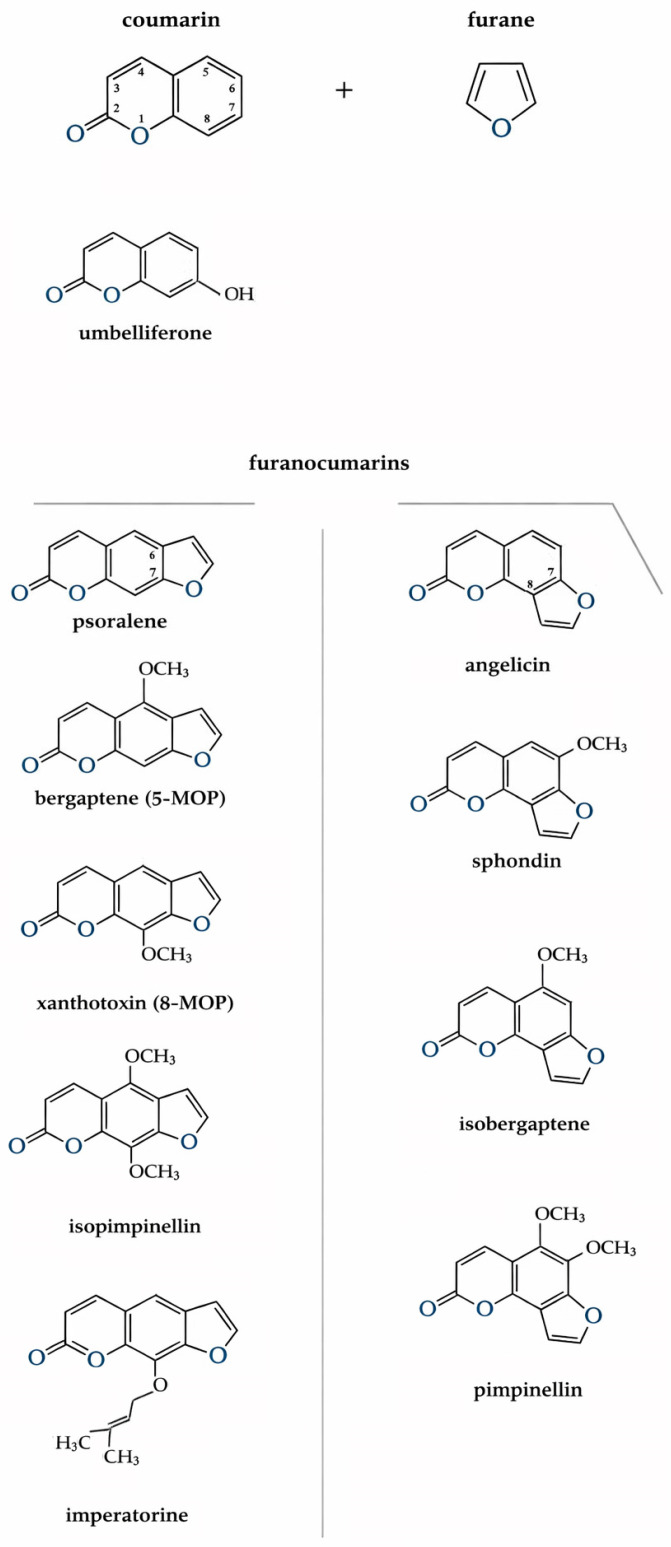
Schematic representation of furanocoumarin formation. Coumarin and furanocoumarins presented in the research.

Fouranocoumarins are a large class of natural organic compounds derived from coumarin, in which the coumarin nucleus is fused with a furan ring. It is precisely this furan ring that is responsible for the photosensitizing activity of these compounds; its substitution leads to a loss of this property. Depending on the position of the rings, furanocoumarins are divided into two structural types: linear (6,7-furanocoumarins), such as psoralen and derivatives, and angular (7,8-furanocoumarins), such as angelicin and derivatives [6] (Figure 2). Most hogweeds contain about 15 coumarins, including psoralen, xanthotoxin (8-MOP or methoxalen), bergapten (5-MOP), umbelliferone, isopimpinellin, imperatorin, and angelicin, sphondin, isobergaptene and pimpinellin. These coumarins are common to many *Heracleum* spp. [38]. In plants of the *Heracleum* genum, coumarin compounds accumulate in essential oil canals or cavities found in fruits, leaves, stems, roots, floral parts, and trichomes. This localization explains why even brief contact with the plant’s surface, especially areas covered in trichomes, can cause rapid photochemical burns [62]. The quantity and composition of furanocoumarins vary throughout the vegetation period and between different organs. Their concentration is highest at the beginning of stem elongation, decreases during budding and flowering, and then rises again as fruits mature. In the roots, maximum furanocoumarin levels are observed during fruit formation [63]. Studies have shown that mature and green seeds of *H. sosnowskyi* contain the greatest amount of these compounds 13.5 and 11.5 mg per gram of air-dried mass, respectively, with almost all concentrated in the essential oil canals of the fruit coats (11.8–29.0 mg/g) [63,64,65]. In plants, furanocoumarins act as secondary metabolites with protective functions. They possess antibacterial, antiviral, and antifungal activity. Their phototoxic properties protect the plant from herbivores and insects, while their allelopathic effects suppress the growth of other plant species giving hogweed invasive advantage [63,66].

### 5.2. Extraction Methods for Furanocoumarin Recovery from H. sosnowskyi

The extraction of furanocoumarins from *H. sosnowskyi* from fresh material is optimal. Furanocoumarin crystals are found on the surface of trichomes and other epidermal cells [62]. Mechanical impact during storage, pre-washing, freezing and grinding can damage the plant’s outer layer and lead to significant furanocoumarin loss. The efficiency of extraction is increased by one to three orders of magnitude when plant material is immersed in hot water [67]. Different solvents are used to extract coumarins from plant materials. These include water (H_2_O) and organic solvents such as ethanol (EtOH), methanol (MeOH), ethyl acetate (EtOAc), chloroform, diethyl ether, petroleum ether and hexane, as well as mixtures thereof [68]. Pyridine, compared to conventional extraction with organic solvents at room temperature, acts as a mild polar basic solvent, simultaneously increasing the solubility of coumarins and reducing their binding to other components of the plant material [69]. The following extraction methods are employed: maceration (infusion at room temperature); Soxhlet extraction (continuous boiling with solvent recovery); and ultrasonic or microwave extraction. The resulting extract was filtered and concentrated under reduced pressure using a rotary evaporator. To purify the coumarin mixture from accompanying substances, including fats, waxes, pigments, and essential oil components, the concentrated extract or the dry residue obtained after solvent removal was treated with nonpolar organic solvents such as chloroform, diethyl ether, or petroleum ether. The organic phase containing lipophilic impurities was removed, while the coumarins remained in the polar residue for further purification. Water-soluble impurities and acidic components were subsequently removed by washing with alkaline or salt solutions (e.g., NaOH or Na_2_CO_3_), followed by drying of the organic phase over anhydrous sodium sulphate(Na_2_SO_4_) [68,70]. Other methods include solid-phase extraction, column chromatography on silica gel with gradient elution (hexane/ethyl acetate or chloroform/methanol), thin-layer chromatography (TLC) and high-performance liquid chromatography (HPLC), including reverse-phase mode (RP-HPLC). Identification of the obtained components is carried out using a combination of physicochemical methods: ultraviolet spectroscopy (absorption maxima in the range of 200–350 nm); NMR spectroscopy (^1^H, ^13^C NMR) to establish structure, including identification of methoxyl groups; and mass spectrometry to determine molecular weight and study fragmentation. Comparison is made with certified standard samples [70].

### 5.3. Analysis of Research on the Extraction of Furanocoumarins from H. sosnowskyi Is Presented Here

Among the reviewed studies, an interesting report by Kulikov et al. describes the development of liposomes containing 8-methoxypsoralen (8-MOP) and 5-methoxypsoralen (5-MOP), as well as their photocytotoxicity in mouse fibroblast culture L929 (Table 1) [71]. Lecithin and cholesterol were used to form liposomes, providing a lipid bilayer capable of encapsulating the hydrophobic psoralen molecules. The highest in vitro phototoxicity was observed for liposomes containing fraction HSf-2.2, isolated from *H. sosnowskyi* and enriched with 5-MOP and 8-MOP. The cytotoxic effect was directly associated with apoptosis induction, primarily mediated through photoinduced covalent DNA cross-link formation. Unlike conventional photosensitizers such as phthalocyanines, liposomal psoralen does not generate significant amounts of reactive oxygen species (ROS). This advantage means that furanocoumarins can be used as an alternative to photodynamic therapy (PDT) when tumour cells become resistant to oxygen-dependent photosensitisers in hypoxic conditions [72]. A comparative analysis revealed that liposomes containing 5-MOP and 8-MOP displayed reduced dark toxicity in vitrowhen compared to photosensitiser chlorin e6 (Ce6). Furthermore, these liposomes maintained both stability and photoactivity for up to eight weeks when stored at +12 °C to +20 °C. Hybrid systems combining furanocoumarins and porphyrins, with liposomal delivery as a key factor enhancing efficacy and bioavailability, may represent a promising strategy for developing next-generation photosensitizers [73].

In the study by Mishina et al. (2015), the content of furanocoumarins in seeds and seed coats of *H. sosnowskyi* before and after stratification was analysed, along with the effects of seed extracts on radicle and hypocotyl growth in seedlings of various plant species (Table 1) [63]. An extract concentration of mg fruits/mL agar was shown to significantly inhibit or completely suppress the growth of both grasses (*Festuca pratensis*, *Lolium multiflorum*, *Lolium perenne* and *Phleum pratense*) and legumes (*Trifolium pratense* and *Trifolium repens*). Comparative phytotoxicity assays of individual furanocoumarins revealed that angelicin exhibited the strongest inhibitory activity, with EC_50_ values of 12 and 11 μg/mL for *Lactuca sativa* radicle and hypocotyl growth, respectively. By contrast, bergapten, methoxsalen and imperatorin exhibited markedly lower phytotoxicity, with EC_50_ values only being reached at concentrations above 200 μg/mL. The authors concluded that the high allelopathic properties of angelicin, combined with the productivity of *H. sosnowskyi* seeds, contributes to the success of its invasive spread [63].

Punegov et al. investigated the juice of *H. sosnowskyi* extracted from aerial plant parts before and after cavitation treatment [74]. Native plant juice contained methoxsalen, isopimpinellin, and angelicin (Table 1). Following electro-discharge cavitation, these furanocoumarins were completely degraded, forming a range of compounds with aromatic and non-aromatic structures not typical for native *H. sosnowskyi* juice. Identified degradation products included 2-phenylethanol, 3-phenylpropanol, 2-hydroxy-5-methylacetophenone, 2,3-butanediol, and benzyl alcohol. Among the organic acids detected, lactic (55.29%) and succinic acids (23.29%) predominated. The authors therefore proposed that cavitation-treated *H. sosnowskyi* juice could serve as a potential stimulant of seed germination and plant growth, as well as a natural preservative for silage and haylage production [74].

A mixture of furanocoumarins obtained by Politowizh et al. from *H. sosnowskyi*, *H. mantegazzianum*, and *H. persicum* (Table 1) exhibited moderate antimicrobial activity at a concentration of 70 μg/mL compared with tetracycline or nystatin at 35 μg/mL [75]. The most susceptible microorganism was *Streptococcus agalactiae*, whose growth was inhibited by 35–41%. Extracts of *H. persicum* and *H. sosnowskyi* demonstrated comparable efficacy against *Escherichia coli* and *Pectobacterium atrosepticum* (26–37%), while the lowest activity was observed against *Staphylococcus aureus*. Extracts from all *Heracleum* species tested were inactive against spore-forming *Bacillus subtilis* and coccoid *Staphylococcus pseudintermedius*. These data are consistent with previous reports describing moderate antimicrobial activity of *Heracleum* extracts against Gram-positive bacteria and yeasts, though substantial differences in effectiveness among species likely reflect variations in their bioactive compound composition [75].

Among reviewed studies, three furanocoumarins, methoxsalen, angelicin, and bergapten, wereconsistently identified (Table 1). These compounds possess notable therapeutic potential, including antitumor activity [43,44], and have long been applied in dermatology for the treatment of psoriasis and vitiligo within PUVA therapy [76,77]. Angelicin is considered the most promising candidate for the development of safer photochemotherapeutic agents. Its advantage lies in ability to form monofunctional DNA adducts without generating interstrand cross-links, thereby substantially reducing photomutagenic and carcinogenic risks compared with linear bifunctional psoralens such as methoxsalen and bergapten, which form double covalent bonds with both DNA strands [78].

Bergapten exhibits anti-inflammatory properties by inhibiting pro-inflammatory cytokines [79]. In vitro and in vivo studies using periodontitis models have shown that psoralen and angelicin have antibacterial activity against *Porphyromonas gingivalis*. In addition, these substances have been found to stimulate osteogenesis in these models [80]. Current research trends are looking at targeted therapies in which furanocoumarins can be used to create effective drugs with minimal toxicity [71].

In addition to its medicinal uses, *H. sosnowskyi* extract, which is rich in furanocoumarins, may serve as a promising basis for the development of multifunctional biopesticides. These biopesticides have the advantage of combining insecticidal, fungicidal and antibacterial properties, a fact supported by extensive data on the broad biological activity of coumarin derivatives [81].

**Table 1:** Extraction of furanocoumarins from *H. sosnowskyi*.

Raw Material	Stems Collected	Leaves and Young Shoots Collected During the Flowering Stage	Mature Fruits Collected in August–September in Belarus and Russia; Fruit Coats Before Stratification Were Used	Fruits Collected in August in Wrocław, Poland	Stems, Leaves and Petioles Collected During the Flowering Stage	Leaves Collected in Komi Republic (Inta) and Novgorod Region (Borovichi), Russia		Stems, Leaves and Petioles Collected During the Flowering Stage	Leaves Collected
Extraction technique	1. The dried and ground material was extracted with chloroform for 24 h. 2. The extract was filtered and evaporated to dryness. 3. The residue was treated with 10% alkali solution and heated for 5 min. 4. The coumarins were re-extracted with chloroform, washed with 5% Na_2_CO_3_, the phases were allowed to separate, and the organic layer was collected, dried over anhydrous Na_2_SO_4_, filtered, and evaporated to dryness. 5. The resulting residue was dried at 70 °C to a constant weight.	1. The dried and ground material was extracted with a 70% ethanol at 98 °C for 3 h. 2. The extract was cooled and vacuum filtered. 3. The filtrate was dried by freeze-drying at a temperature of −55 °C for 40 min.	1. The dried and ground material was extracted with methanol for 48 h. at room temperature and treated twice with ultrasound for 1 min. 2. The extract was filtered through filter paper No. 1, centrifuged at 3000 rpm for 10 min., and the supernatant was collected. 3. A Shim-Pack VP-ODS column was used to separate furanocoumarins in the supernatant.	1. The dried and ground material was extracted with hexane at room temperature for 24 h. 2. The extract was filtered through filter paper No.1. 3. The solvent was removed from the extractsunder reduced pressure using a rotary evaporator at a water bath at 40 °C. 4. To remove residual volatile components (essential oils), the residues were freeze-dried for 24 h.	1. The raw materials were crushed, and the juice was squeezed shortly after collection. 2. The juice was extracted twice with chloroform, and the combined extracts were concentrated to a resinous residue under reduced pressure using a rotary evaporator. 3. Nonpolar lipids were removed by treatment with hexane at 50 °C, yielding a polar residue. 4. The polar fraction was derivatized with a silylating mixture of pyridine, TMCS, and BSTFA (2:1:1) and sonicated for 5 min.	I. Ethanol extraction: 1. The dried and ground material was extracted with 95% ethanol for 4 h in a Soxhlet apparatus.	II. Sublimation technique: 1. The dried and ground material was mixed with magnesium oxide in a 1:1 ratio and heated at 100 °C for 25 min. 2. Sublimed compounds were condensed as a crystals using round-bottom flask filled with ice. 3. The crystals were treated with a 10% alcoholic KOH and heated in a water bath for 5 min. 4. Coumarins were precipitated by the addition of 10% HCl, filtered, and weighed.	1. The raw materials were crushed, and the juice was squeezed out 1 h after collection. 2. The juice was extracted twice with chloroform (4:1, chloroform:juice) for 24 h at 25 °C under constant stirring. 3. The organic phase was separated and evaporated to dryness under reduced pressure using a rotary evaporator at a water bath at 50 °C. 4. The residue was treated with 10% NaOH solution at 60–70 °C, followed by re-extraction of furanocoumarins with chloroform. 5. The combined chloroform extracts were washed with 5% Na_2_CO_3_ solution, dried over anhydrous Na_2_SO_4_ for 24 h, filtered, and evaporated to constant weight. 6. The dry extract was successively dissolved in acetonitrile and absolute ethanol with heating and sonication, cooled to −18 °C to induce precipitation 7. The precipitate was dissolved in benzene and used for column chromatography.	1. The dried and ground material was extracted using solvents of different polarity (water, methanol, acetone, or hexane) under microwave heating at 50 °C for 10 min. 2. Hexane was selected as the optimal solvent, and the extraction was performed at 70 °C for 10 min using 2 mL of hexane and 1 mL of water per 0.1 g of material 3. The resulting extracts were filtered, purified by solid-phase extraction on a Strata Eco-Screen sorbent, and analysed by GC–MS.
Main compounds, extraction yield	coumarin mixture total yield—0.8%	metaxolene 1.15% bergapten 1.04% umbelliferone 0.83% angelicin 0.63% sphondin 0.35% (not found in leaves) total yield—4.00%	angelicin 11.8–29.0 mg/g bergapten 5.0–7.1 mg/g imperatorin 0.4–7.5 mg/g methoxalen 0.5–8.7 mg/g (depending on the place of collection)	isopimpinellin 37.81% isobergaptene 21.15% pimpinellin 18.52% bergaptene 15.20% angelicin 6.02% imperatorin 0.66% psoralene 0.52% methoxsalen 0.09%	methoxalen 12.29% angelicin 12.00% isopimpinellin 1.72% total yield—26% (Three furocoumarins were found in the polar fraction of lipids.)	Komi Republic: total yield—22.6% Novgorod region: total yield—5.9%	Komi Republic: total yield—0.33% Novgorod region: total yield—0.1%	8-methoxypsoralen (methoxalen) 1332.7 mg/L (1.333 mg/g) 5-methoxypsoralen (bergaptene) 34.2 mg/L (0.0342 mg/g)	angelicin 2.281 mg/g methoxalen 0.762 mg/g bergapten 0.314 mg/g psoralen 0.146 mg/g total yield 3.5 mg/g (Pure angelicin and methoxalen were successfully isolated.)
Detection technique	Thin-layer chromatography (TLC)	Paper chromatography	HPLC-UV using an LC-20AD system (Shimadzu, Japan) with a SPD-M20A UV detector and a Shim-pack VP-ODS (C18) column: methanol–water gradient, detection at 280 nm.	GC-MS using Saturn 2000 MS (Varian Chrompack, USA) and TRACE DSQ (Thermo, USA) equipped with a ZB-5 column.	GC–FID on a Kristall-2000M (Chromatec, Russia) with identification by GC–MS on a TRACE DSQ system using a TR-5MS column.	Lactone test(qualitative detection); UV–Vis spectrophotometry at 360 nm (quantitative determination).		TLC (UV 365 nm) followed by identification using UV–Vis spectrophotometry, HPLC–UV (Gilson-Rainin 307, Kromasil C18), and ^1^H/^13^C NMR (JEOL JNM ECX-400).	GC–MS identification and GC–FID quantification after solid-phase extraction, using an Elite-5MS capillary column (PerkinElmer).
Application			Angelicin showed the strongest inhibitory activity, with EC_50_ values of 12 µg/mL for root growth and 11 µg/mL for hypocotyl growth of *Lactuca sativa*.	A furanocoumarin mixture exhibited moderate antimicrobial activity at 70 µg/mL. The highest sensitivity was observed for *S. agalactiae* (35–41% growth inhibition), with comparable effects against *E. coli* and *P. atrosepticum* (26–37%). Minimal activity was noted against *S. aureus*, and no activity was detected against spore-forming *B. subtilis* or *S. pseudintermedius*.	After electrodischarge cavitation treatment, furanocoumarins in the juice were degraded, forming mainly 2-hydroxypropionic (55.29%) and succinic acids (23.29%), which can be used as seed germination stimulants, plant biostimulants, and preservatives for silage and haylage.			Liposomal formulations of 5-MOP and 8-MOP have been developed for anticancer applications and exhibit lower dark toxicity in vitro compared with the conventional photosensitizer chlorin e6 (Ce6).	
Ref.	[82]	[83]	[63]	[75]	[74]	[15]		[71]	[84]

## 6. *H. sosnowskyi* Is a Source of Essential Oil

### 6.1. Characteristics of Essential Oil in H. sosnowskyi and Methods of Extracting It from H. sosnowskyi

The essential oil of *H. sosnowskyi* is primarily composed of octyl acetate, saturated aliphatic esters, 1-octanol, octanal, and various terpenes [11]. Essential oil production occurs through steam distillation. The plant’s secretory structures are destroyed by superheated steam, which also releases volatile aromatic compounds. After the steam mixture has condensed, the essential oil separates from the aqueous phase due to differences in polarity and density. The seeds can be processed using cold pressing [85]. Alternative extraction methods include solvent extraction using petroleum ether, hexane, or ethanol, as well as supercritical CO_2_ fluid extraction. These approaches generally provide higher yields, resulting in the production of a total lipophilic extract. The extraction method significantly affects the composition of the final product. In steam distillation and cold pressing, furanocoumarins are largely absent from the resulting oil, remaining in the distillation residue due to their low volatility and high boiling points. Conversely, solvent extraction particularly with nonpolar solvents facilitates the efficient extraction of furanocoumarins, rendering the final product potentially phototoxic [86,87]. The qualitative and quantitative composition of the essential oil varies substantially. This depends on the plant organ, vegetative phase, geographical origin, soil and climatic conditions. It also depends on the extraction technique used [11]. The essential oil content has been reported to reach up to 0.5% in roots, 0.65–0.75% in leaves, 0.52–0.94% in flowers, and between 1% and 10% in fruits [49]. Hogweed fruits harvested at the wax ripening stage are of the greatest practical interest, as the major oil reserves are localised within the secretory canals of fruit coats. Harvesting should be performed during overcast weather to minimise the loss of volatile components through evaporation [27]. Prolonged storage of fruits is not recommended, as it leads to a significant decrease in essential oil content due to volatilization [88].

### 6.2. Analysis of Research on the Extraction of Essential Oil from H. sosnowskyi Is Presented Here

Tkachenko (2010) reported significant differences in the composition of essential oils obtained from fruits of *H. sosnowskyi* growing in its natural range compared with those of introduced populations (Table 2) [26]. Cultivation under new soil and climatic conditions led to the activation of terpene biosynthesis in the fruit essential oil, which is likely an adaptive mechanism enhancing plant survival under cold environmental conditions. Quantitative ratios of individual components were also altered. The author demonstrated that the composition of *H. sosnowskyi* fruit essential oil depends on extraction method, collection year, and plant vegetative period, which complicates the use of chemical data for chemosystematic purposes [26].

Mishina et al. (2015) investigated the essential oils of invasive *H. sosnowskyi* and native species (*H. lescovii*, *H. asperum*, *H. dissectum*, *H. hirtum*) collected from various regions of Russia and Belarus (Table 2) [14]. The volatile fractions of fruits were evaluated for phytotoxicity against *Lactuca sativa* seedlings. Volatile compounds of *H. sosnowskyi* fruits showed the most pronounced inhibitory activity, suppressing radicle and hypocotyl growth by 12–40% and 8–42%, respectively, depending on the collection site, while seed germination remained unaffected. Native species exhibited weaker effects: *H. lescovii* and *H. asperum* inhibited radicle growth by 17% and 12%, *H. hirtum* inhibited hypocotyl growth by 14%, and *H. dissectum* showed no significant activity. Based on GC–MS analysis, octanal was identified as the main phytotoxic compound. In bioassays, the growth of radicles and hypocotyls of *Lactuca sativa* was inhibited by octanal at concentrations of 20 µg/mL for radicles and 9 ng/cm^3^ for hypocotyls, with the inhibition rate being 50%. These findings suggest that the strong phytotoxicity of *H. sosnowskyi* volatiles, particularly octanal, may contribute to invasiveness by providing competitive advantages through allelopathic effects [14].

In a subsequent study [89], volatile compounds from *H. sosnowskyi* fruits demonstrated strong antifungal activity. These compounds were able to inhibit mycelial growth by up to 67%. The individual compounds tested were octanol, octanal and trans-2-hexenal. Octanol exhibited the highest efficacy against two races of *Fusarium oxysporum* f. sp. *lycopersici* (FOL), with EC50 values of 8.1 ng/mL (race 1) and 9.3 ng/mL (race 2). Soil treatment with trans-2-hexenal and octanol significantly reduced the severity of infection and the density of conidia of both pathogen races. Octanal only inhibited race 2. These results highlight the potential of volatile compounds, particularly octanol and trans-2-hexenal, as environmentally friendly biofumigants for controlling *Fusarium* wilt [89].

Synowiec and Kalemba (2015) extracted essential oil from *H. sosnowskyi* fruits and evaluated inhibitory effects on the germination of weed and maize seeds (Table 2) [90]. The most susceptible species were *Bromus secalinus* and *Amaranthus retroflexus*, for which 0.63 and 0.67 g/L of essential oil inhibited 50% of seed germination (ED_50_), respectively. *Avena fatua* and *Centaurea cyanus* exhibited moderate sensitivity, with ED_50_ values of 1.26 and 1.68 g/L, respectively. The least sensitive were *Echinochloa crus-galli* and *Zea mays*, requiring 2.37 and 2.48 g/L to reach ED_50_. Maize seeds remained tolerant to concentrations up to 0.6 g/L, and 20% of seeds germinated even at 7.2 g/L. These results indicate that *H. sosnowskyi* essential oil has potential as a natural selective herbicide [90].

Politowizh et al. (2017) reported that essential oils extracted from *H. sosnowskyi*, *H. mantegazzianum*, and *H. persicum* fruits exhibited no antimicrobial activity against Gram-positive bacteria (*Staphylococcus aureus* PCM 2054, *S. pseudintermedius* KP-Spi1, *Streptococcus agalactiae* KP-Sag1, *Bacillus subtilis* PCM 1949), Gram-negative bacteria (*Escherichia coli* PCM 2057, *Pectobacterium atrosepticum* IOR-1826), or the yeast *Candida albicans* KP-Ca1 [75].

However, Lisovenko et al. (2025) demonstrated strong antifungal activity of *H. sosnowskyi* fruit essential oil against the yeast-like fungus *Candida albicans* (Table 2) [91]. Fungistatic effects were observed at an oil concentration of 0.39%, while complete culture inhibition occurred at 1.50% emulsion. The antifungal efficacy of *H. sosnowskyi* oil was comparable to that of fennel (*Foeniculum vulgare*) fruit essential oil [92]. These results support the potential use of *H. sosnowskyi* essential oil as a natural fungicidal agent for applications in medicine, veterinary science, and agriculture [91].

The discrepancy in antimicrobial results between the studies of Politowizh et al. [75] and Lisovenko et al. [91] appears to be methodological. Politowizh et al. employed the agar disc diffusion method, in which the hydrophobic and volatile essential oil poorly diffuses in the water-saturated agar medium, potentially leading to false negatives [75]. In contrast, the same authors reported antimicrobial activity for furanocoumarin fractions dissolved in DMSO, which facilitated diffusion from the paper disc and established an effective inhibition gradient. Lisovenko et al. applied the broth microdilution method, which eliminates diffusion limitations and allows direct contact between the test compound and microorganisms throughout the medium, providing a more accurate assessment of biological response [91].

**Table 2:** Extraction of essential oil from *H. sosnowskyi* fruits.

Raw Material	Fruits Collected from Naturally Growing Plants in North Ossetia and Leningrad Region, Russia	Fruits Collected in August–September from Plants Growing in Russia and Belarus	Fruits Collected from the Main Umbels of Multiple Plants Grown in a Monoculture in Garlica Murowana, Near Krakow, Poland	Fruits Collected in August in Wrocław, Poland	Whole Fruits Collected in August in Yaroslavl, Russia	Fruits Collected in Late August-Early September in the Perm Region, Russia
Extraction technique	Hydrodistillation using a Deryng apparatus. 1. The air-dried and ground fruits were placed in a round-bottom flask together with distilled water containing 20–30% NaCl. 2. The flask was heated for 2 h after reaching the boiling point, and the vapours were condensed using a water-cooled condenser. 3. To evaluate the preservation of highly volatile components, essential oil isolation was additionally performed using a modified apparatus in which the reaction flask was placed in a microwave unit, followed by conventional condensation through a reflux water condenser. 4. After 120 min, the essential oil was collected in 2.5 mL vials and stored at −15 °C	Volatile compound extraction (Headspace sampling) 1. Thenonstratified fruits were placed in a sealed glass vial (23 × 75 mm, GRACE, Japan). 2. The vial was incubated for 1 h to allow volatile compounds to equilibrate in the headspace. 3. Headspace collection: 200 μL of the headspace gas was sampled for analysis.	The fruits were dried and ground using an electric grinder. Essential oil was obtained by hydrodistillation using a Clevenger-type apparatus.	Hydrodistillation using a Deryng apparatus. 1. The dried and ground fruits were placed in a round-bottom flask together with distilled water. 2. The flask was heated for 2 h after reaching the boiling point, and the vapours were condensed using a water-cooled condenser. 3. After 120 min, the essential oil was collected in 2.5 mL vials and stored at −15 °C	Essential oil was obtained from whole air-dried fruits one month collected by Soxhlet extraction. 1. The fruits were extracted in a Soxhlet apparatus with pure petroleum ether (4:1, solvent:fruits) in five consecutive cycles at 40–70 °C for 40 min each. 2. The solvent was then evaporated to a 2:1 volume ratio of petroleum ether to essential oil. 3. The residue was mixed with vaseline oil (1:1, essential oil in petroleum ether:vaseline oil), and petroleum ether was removed by evaporation at atmospheric pressure.	1. The dried and ground fruits were placed in a wide-necked flask connected to a steam generator. 2. The vapours of essential oil and water were condensed in a water-cooled condenser and collected in a separatory funnel equipped with water and separating funnel equipped with a water and air outlet. 3. The aqueous phase and essential oil were separated in the separating funnel; distillation was continued for at least 2 h after boiling commenced in the steam generator.
Main compounds, extraction yield	1. Octyl acetate 55.0–63.0 2. Hexyl 3-methylbutanoate 9.5–14.0 3. Hexyl butanoate 5.1–7.7. 4. Octyl 2-methylpropanoate 2.1–6.7 5. Octyl 3-methylbutanoate 2.0–3.4 6. 1-Octanol 0.8–3.3 not found in fruits collected in North Ossetia: Hexyl 2-methylpropanoate 3.9–7.3 α-Pinene 0.4–2.9 Octanal 0.5–1.2	1. Octyl acetate 5.8–48.9 2. Hexyl 2-methylbutanoate 2.2–17.0 3. Hexyl 2-methylpropanoate 1.3–19.7 4. n-Hexyl acetate 0.8–13.7 5. 1-Octanol 0.2–9.1 6. Octanal 0.6–4.9 7. γ-Terpinen < LOD-3.5	1. Octyl acetate 39.5 2. Hexyl 2-methylbutanoate 14.4 3. 1-Octanol 8.6 4. Hexyl 2-methylpropanoate 6.0 5. Hexyl butanoate 5.4 6. Octyl 2-methylbutanoate 4.0 7. Hexyl 3-methylbutanoate 2.6 8. Octanal 0.7 total yield 5.1% (*v*/*w*)	1. Octyl acetate 43.44 2. Hexyl butanoate 11.51 3. Decanal 9.51 4. Hexyl 2-methylbutanoate 6.47 5. Hexyl 2-methylpropanoate 3.85 6. Butyl butanoate 2.26 7. 1-Octanol 2.15 total yield 3.6–4.5% (*v*/*w*)	1. Octyl acetate 27.575 2. Terpene 14.671 3. Octyl 3-methylbutanoate 7.568 4. Hexyl butanoate 6.891 5. Octanal 5.225 6. Hexyl hexanoate 3.375 7. Hexyl acetate 3.297 8. 1-Octanol 3.257 total yield 3.4% (*v*/*w*)	1. Octyl acetate 37.48 2. Hexyl 2-methylbutanoate 13.41 3. Octyl 2-methylbutanoate 7.47 4. Hexyl butanoate 6.45 5. Octyl 2-methylpropanoate 4.64 6. 1-Octanol 4.60 7. Octyl butanoate 3.37 total yield 4% (*v*/*w*)
Detection technique	GLC–FID using a Tsvet-500 gas chromatograph (Russia) equipped with a DB-1 capillary column with temperature programming.	HS-GC-MS using GC-MS-QP 2010 Plus system (Shimadzu, Japan) equipped with an EQUITY-5 column; identification by NIST/Wiley libraries and authentic standards, quantification by calibration curves.	GC-FID and GC-MS using Trace GC Ultra-DSQII system (Thermo Electron) equipped with a Rtx-1 MS column; identification by retention indices and mass spectra compared (NIST09, Wiley 275, MassFinder 4.1); quantification by FID.	GC-MS using a Saturn 2000 MS (Varian Chrompack, USA)equipped with a ZB-5 column; identification by retention indices and NIST14 mass spectra; quantification by GC-FID (Agilent 7890N).	GC-FID using a Crystallux 4000M system (Meta-chrome, Russia) equipped with a DB-624 capillary column; identification by retention times; quantification by internal normalisation.	GC–MS using an Agilent 7890Aequipped with a HP-5ms capillary column; samples diluted in 95% ethanol (1:50); identification by full-scan mass spectra.
Application		Octanal inhibited 50% of lettuce root and hypocotyl growth at concentrations of 20 and 9 ng/cm^3^, respectively. Octanol showed the highest efficacy against two *Fusarium* strains, with the lowest EC_50_ values of 8.1 ng/mL (strain 1) and 9.3 ng/mL (strain 2). Soil treatment with trans-2-hexenal and octanol minimised tomato infection, highlighting their potential as eco-friendly biofumigants against *Fusarium* wilt.	Essential oil from fruits inhibited seed germination of weeds and maize. *Bromus secalinus* and *Amaranthus retroflexus* were most sensitive, with 0.63–0.67 g/L causing 50% inhibition, while *Echinochloa crus-galli* and *Zea mays* were least sensitive (ED_50_ 2.37–2.48 g/L). These results highlight the potential of *H. sosnowskyi* oil as a basis for selective natural herbicides.	Essential oil from fruits showed no antimicrobial activity against Gram-positive bacteria (*S. aureus*, *S. pseudintermedius*, *S. agalactiae*, *B. subtilis*), Gram-negative bacteria (*E. coli*, *P. atrosepticum*), or fungi (*C. albicans*).	To improve technological properties, the essential oil from fruits was mixed 1:1 with vaseline and the fraction boiling below 85 °C was removed. The resulting rubber showed a 23% increase in frost resistance at −45 °C compared to rubber plasticized with industrial oil, while maintaining its mechanical properties.	Essential oil from fruits showed strong antifungal activity against the yeast-like fungus *Candida albicans*. A fungistatic effect was observed at 0.39% concentration, while complete culture death occurred at 1.50% emulsion.
Ref.	[26]	[14,89]	[90]	[75]	[88,93]	[91]

Pavlov and Solovyov investigated *H. sosnowskyi* fruit essential oil with a high content of fatty acid esters and proposed use it in the production of frost-resistant rubber materials (Table 2) [88]. The oil yield from fresh biomass was 3.4% (*w*/*w*); however, after one year of storage, extraction with light petroleum ether was ineffective, yielding only trace amounts. To increase extraction efficiency from stored material, a 1:1 acetone–benzene solvent mixture was used. Reanalysis revealed a reduction in terpenes, octanal, hexyl acetate, and octyl isobutyrate, as well as the complete disappearance of 1-hexanol, accompanied by increased proportions of hexyl butyrate, hexyl caproate, octyl isobutyrate, and several unidentified compounds. The total content of identified fatty acid esters increased by 2.3%. To improve processing properties, the essential oil was mixed with vaseline oil (1:1), and fractions boiling below 85 °C were removed. Rubber modified with this blend showed a 23% increase in frost resistance at –45 °C compared with rubber plasticized with industrial oil, while maintaining mechanical strength. The improved cold resistance is attributed to the high content of fatty acid esters (57.5% *w*/*w*) in *H. sosnowskyi* oil, which acts not only as a softener but also as an efficient plasticizer, facilitating rubber granulation and reducing cracking of road surfaces at low temperatures [88,93].

Among all studies, octyl acetate (5.8–63.0%) consistently dominated the essential oil composition. C_6_–C_8_ fatty acid esters such as hexyl butanoate (5.1–11.5%), hexyl 2- or 3-methylbutanoate (2.2–17.0%), and octyl 2- or 3-methylbutanoate (2.0–7.5%) constituted major components, while 1-octanol (0.2–9.1%) and, less frequently, octanal/decanal (0.5–9.5%) were present in moderate amounts. Terpenes (including γ-terpinene and α-pinene) were detected in minor quantities (<LOD–14.6%) but contribute synergistically to the insecticidal and fungicidal effects of the mixture [14,26,75,88,90,91].

The high content of esters makes *H. sosnowskyi* essential oil a valuable raw material for chemical industries [88,93]. A correlation has been established between the component composition and biological activity: aldehydes (octanal, decanal) and alcohols (1-octanol) are responsible for pronounced antifungal and antibacterial effects [89,91], while the complex of volatile compounds exhibits insecticidal and allelopathic properties [14], with promising applications as biofumigants [94] and natural herbicides [90]. The identified antiseptic and anti-inflammatory properties of the constituents also open prospects for pharmaceutical use as a basis for topical antimicrobial formulations, anti-inflammatory ointments, and wound-healing preparations.

## 7. Conclusions

The main problem in processing *H. sosnowskyi* is the significant variability in the composition and concentration of the target products, furanocoumarins and essential oil, which makes it difficult to standardise the technological process. However, in the context of combating invasive species, processing can be viewed not as commercial production, but as a means of utilising biomass, which additionally allows valuable substances to be obtained. The creation of waste-free technological chains is a promising direction. For example, the cellulose residue formed after the extraction of biologically active compounds can be used to produce fuel pellets or cellulose. This solution would significantly reduce the volume of harmful production waste. To successfully combat hogweed, effective government programmes are needed to map and monitor the spread of invasive species, restore damaged ecosystems, reclaim land and encourage agricultural use of abandoned land. The processing of invasive hogweed thickets can become an element of sustainable natural resource management. This combines environmental safety with practical benefits. But its effectiveness depends on government support and a systematic approach.

## Data Availability

No new data were created or analysed in this study.
